# Models of care for the management of alcohol use disorder in general hospital settings and transition to the community: a scoping review

**DOI:** 10.1093/alcalc/agag037

**Published:** 2026-07-06

**Authors:** Julia M A Sinclair, Sofia Hemrage, Nicola J Kalk, Georgia Foote, Melinda King, Julia Morris, Seilin Uhm, Amy Kitajima, Paul S Haber, Thomas Phillips

**Affiliations:** School of Clinical and Experimental Sciences, Faculty of Medicine, University of Southampton, Academic Centre, College Keep, 4-12 Terminus Terrace, Southampton, SO14 3DT, Southampton, United Kingdom; School of Clinical and Experimental Sciences, Faculty of Medicine, University of Southampton, Academic Centre, College Keep, 4-12 Terminus Terrace, Southampton, SO14 3DT, Southampton, United Kingdom; South London and Maudsley NHS Foundation Trust Bethlem Royal Hospital, Monks Orchard Road, Beckenham, Kent BR3 3BX, United Kingdom; Addictions Department, Institute of Psychiatry, Psychology, and Neuroscience, King’s College London, Denmark Hill, London, SE5 8BB, United Kingdom; School of Clinical and Experimental Sciences, Faculty of Medicine, University of Southampton, Academic Centre, College Keep, 4-12 Terminus Terrace, Southampton, SO14 3DT, Southampton, United Kingdom; School of Clinical and Experimental Sciences, Faculty of Medicine, University of Southampton, Academic Centre, College Keep, 4-12 Terminus Terrace, Southampton, SO14 3DT, Southampton, United Kingdom; School of Clinical and Experimental Sciences, Faculty of Medicine, University of Southampton, Academic Centre, College Keep, 4-12 Terminus Terrace, Southampton, SO14 3DT, Southampton, United Kingdom; School of Health Sciences, Faculty of Environmental Life and Health Sciences, Highfield Campus University of Southampton, Southampton, Hampshire SO17 1BJ, United Kingdom; Concord Hospital, Hospital Road, Concord NSW 2139, Australia; Drug Health Services, Royal Prince Alfred Hospital, King George V Building, Missenden Rd, Camperdown, NSW 2050, Australia; Drug Health Services, Royal Prince Alfred Hospital, King George V Building, Missenden Rd, Camperdown, NSW 2050, Australia; Central Clinical School, Sydney Medical School, University of Sydney, 2006, NSW, Australia; Centre for Addiction & Mental Health Research, Allam Medical Building, University of Hull, Hull, HU6 7RX, United Kingdom

**Keywords:** alcohol use disorder (AUD), models of care, consultation liaison, screening, brief intervention, and referral to treatment (SBIRT), protocol implementation, supported diversion

## Abstract

**Background:**

Early detection of alcohol use disorder (AUD) amongst people admitted to general hospitals offers an opportunity for early intervention and accesses to evidence-based care. However, current operational models for the management of AUD in these settings are poorly defined, limiting the ability to assess their impact on individual patient outcomes, treatment effectiveness, or healthcare system efficiency. This scoping review aims to identify and characterize existing models of AUD management within general hospitals.

**Methods:**

A scoping review approach was adopted, including relevant peer-reviewed publications between 1990 and 2025. Studies needed to report on ≥2 care components (systematic screening, brief interventions, medically assisted alcohol withdrawal, relapse prevention initiation, psychosocial interventions, transition to community, provision of training) to be included. Screening and data extraction were performed independently by at least reviewers.

**Results:**

Fifty-one (*n* = 51) records were included, and four distinct models of care were identified (consultation liaison; screening, brief intervention, and referral to treatment; protocol implementation; supported diversion). Models varied in their clinical purpose, target population, and care delivery focus. Within each model, differences in aims, context, and implementation resulted in substantial heterogeneity.

**Conclusion:**

Consultation liaison models provided the most multifaceted care, with a specialist team providing clinical leadership, access to evidence-based interventions, transition to community services, and training of the wider workforce, but rarely described any wider systematic screening for AUD. A consistent observation across all identified models was the limited interface with mental health care, representing a critical gap in current AUD management within general hospitals.

## Introduction

Globally, alcohol consumption is a significant public health concern and an established risk factor for over 200 health conditions (World Health Organization 2024). Hospital admissions resulting from alcohol use disorder (AUD) and alcohol associated harm have escalated globally in recent years (Kast et al. 2025). This has direct implications at the individual (micro-level), organizational (meso-level), and health system (macro-level) level through diminished health-related quality of life, increased morbidity, and mortality, significant challenges for treatment provision and ineffective use of resources (Griswold et al. 2018, Phillips et al. 2021). Hospital-based management of AUD also has an essential role in the wider healthcare pathway, facilitating; secondary prevention, safe management of alcohol withdrawal, timely identification and management of complications (e.g. alcohol related liver disease, Wernicke Korsakoff Syndrome), and onward engagement with specialized treatment services.

More broadly, ‘models of care’ are structured approaches to delivering health services to ensure people ‘get the right care, at the right time, by the right team, and in the right place’ (Agency for Clinical Innovation 2013). The aim is to support integrated high-quality care, as well as ensuring the efficient use of limited resources. Based on these principles, a model of care for people with AUD and associated harm would encompass multidisciplinary, integrated care across providers, disciplines, and services (National Institute for Health and Care Excellence 2017, American Society of Addiction Medicine 2020, National Health and Medical Research Council 2020, Department of Health and Social Care 2025, Sinclair et al. 2026). In a general hospital setting, this may involve initial systematic (universal or targeted) screening for alcohol use, the provision of medically assisted alcohol withdrawal (MAAW), delivery of evidence-based pharmacological and psychosocial interventions for AUD, and transition to onward services. A model of care for the management of AUD and associated harm, can therefore be conceptualized as a complex intervention, made up of a range of components which will vary based on defined need, context, implementation, and setting (Brennan et al. 2019, Skivington et al. 2021).

Embedding sustained addiction and broader AUD care into general hospital settings is universally challenging (Sinclair et al. 2025, Williams et al. 2025). A review of hospital-based addiction consultation care noted that most staff in general hospital settings did not possess the necessary skills to address the multiple needs of individuals with AUD (Englander et al. 2022). However, at the (macro) health system level, effective early intervention, and sustained treatment engagement of people with AUD also has the potential to reduce overall healthcare utilization (Witkiewitz et al. 2018). While pharmacological and psychosocial interventions for AUD have a well-established evidence base within specialist addiction settings, their potential effectiveness in the general hospital setting will be altered by the range of complexity and severity of presentations, as well as organizational variation and context (Gilburt et al. 2015; Scoles et al. 2025; Sinclair et al. 2026). There is an absence of formal, standardized treatment pathways for non-treatment-seeking clinical populations with AUD across general hospital settings, and limited structures for skills training and intervention uptake. The lack of cohesive best practice or policies at the macro (health system) level has implications on individual health outcomes, treatment engagement, and recovery trajectories (Simpson 2004, Roberts et al. 2020). This is especially important in this patient group who range from people who may only need a brief intervention around alcohol consumption, to those with AUD and significant levels of physical and mental health comorbidity. Effective identification and management of AUD does not fit easily within many healthcare systems, often resulting in disjointed and ineffective care (Sinclair et al. 2025). General hospital setting potentially offer the opportunity to identify, engage, and ensure, where appropriate, onward referral for this complex patient group.

This review aims to identify and characterize the range of models reported for the identification and management of AUD within general hospital settings. It is part of a wider programme of research (ProACTIVE) a multidisciplinary, integrated mixed methods programme designed to evaluate the impact, value, and effectiveness of alcohol care teams (ACTs), a hospital-based model of care, at the patient (micro), health system (meso), and policy (macro) levels (National Institute for Health and Care Research 2022).

## Materials and methods

### Study design and protocol

A scoping review was chosen as the appropriate method to map existing models, rather than producing a critically appraised synthesis of each model and their respective outcomes (Munn et al. 2018). We followed a six-stage methodological framework (identification of research question, identification of relevant studies, study selection, data charting, collation, summary, and report of the results) for scoping reviews (Arksey and O’Malley 2005). The protocol was registered prospectively (Foote et al. 2023) and adheres to the Preferred Reporting Items for Systematic reviews and Meta-Analyses extension for Scoping Reviews (PRISMA-ScR) guidelines (Supplementary File S1; Tricco et al. 2018; Foote et al. 2023). A steering group, including the input of experts by experience, guided study design and planning.

### Inclusion criteria and search strategy

Peer-reviewed publications reported in English and published between January 1990, and December 2025 were included. No restrictions were applied to study design, other than that they needed to report the implementation of a model into routine care, rather than a process that was only set up as a component of a research trial. Studies were included if they reported primarily on adult participants (aged 18 years or over), from any country, with an alcohol-related condition (increased alcohol intake or AUD), presenting to a general hospital setting. Records which included models for managing substance use disorders more broadly were included if they reported specifically on the proportion of those with primary AUD.

As this review aimed to define the variety of models of care, rather than focusing on the effectiveness of a specific intervention, studies were included if they reported on at least two care components, based on expert consensus regarding the core components of general hospital AUD care (Public Health England 2019; Phillips et al. 2020): systematic screening, brief interventions/brief advice (BI/BA), MAAW, initiation of pharmacological relapse prevention (RP) treatment, delivery of additional psychosocial interventions (other than BI/BA), transition of patients to community services at discharge, and ongoing provision of training to other teams. Comparators and reported outcome measures were not considered as criteria to determine eligibility for the review. Grey literature was not included.

A comprehensive search strategy was initially developed and further refined following pilot testing against a sample of relevant peer-reviewed publications. The final search syntax (Supplementary File S2) was run in the following databases (December 2025): CINAHL, PubMed, and Cochrane Library. Additional literature was sought through backward citation searching of relevant articles and hand searching relevant journals.

### Study selection, data extraction, and synthesis

The search output was uploaded to Rayyan, and duplicates were removed (Ouzzani et al. 2016). Articles were independently screened at the title and abstract level, and full-text level by at least two reviewers, and inconsistencies across reviewers were resolved through discussion between the authors. The final study selection was uploaded to Mendeley for reference management (Elsevier 2014).

Data extraction was also undertaken by at least two reviewers, and mapping followed an iterative approach. Extraction forms were developed by the team and tested against a sample of relevant papers. Data were extracted for included study characteristics (country, funding, conflicts of interest, study design, setting, aim of model, target population, staffing, hours of operation) and any reported components of care (systematic screening, BI/BA, MAAW, RP initiation, additional psychosocial interventions, transition to community services, and staff training). This was further discussed during steering group meetings involving members with expertise in alcohol care delivery, academic expertise in an area of alcohol-associated harm, as well as experts by experience. Inconsistencies were discussed and a consensus achieved. Where a component of care was not mentioned, it was assumed not to be delivered.

As the aim is to provide an overview of the existing models for the management of AUD in general hospital settings and identify key gaps in treatment, rather than a synthesis of reported outcomes, data were analysed narratively. A key member of the review team (MK) reviewed the findings from a lived experience perspective and drafted her response to the findings (see Supplementary File S5). Given the wide range of study designs and substantial heterogeneity of reporting styles, none of the well-validated quality assessment tools gave an appropriate way of comparing quality across such a range of studies.

The Context and Implementation of Complex Interventions (CICI) framework (Pfadenhauer et al. 2017) recognizes interactions between context, implementation and setting, and how these can be operationalized to understand complex interventions at the micro, meso, and macro levels. The CICI framework is part of the overall methodology of the wider ProACTIVE programme and therefore used to guide the discussion of this review.

## Results

### Overview of included studies

**Figure 1:**
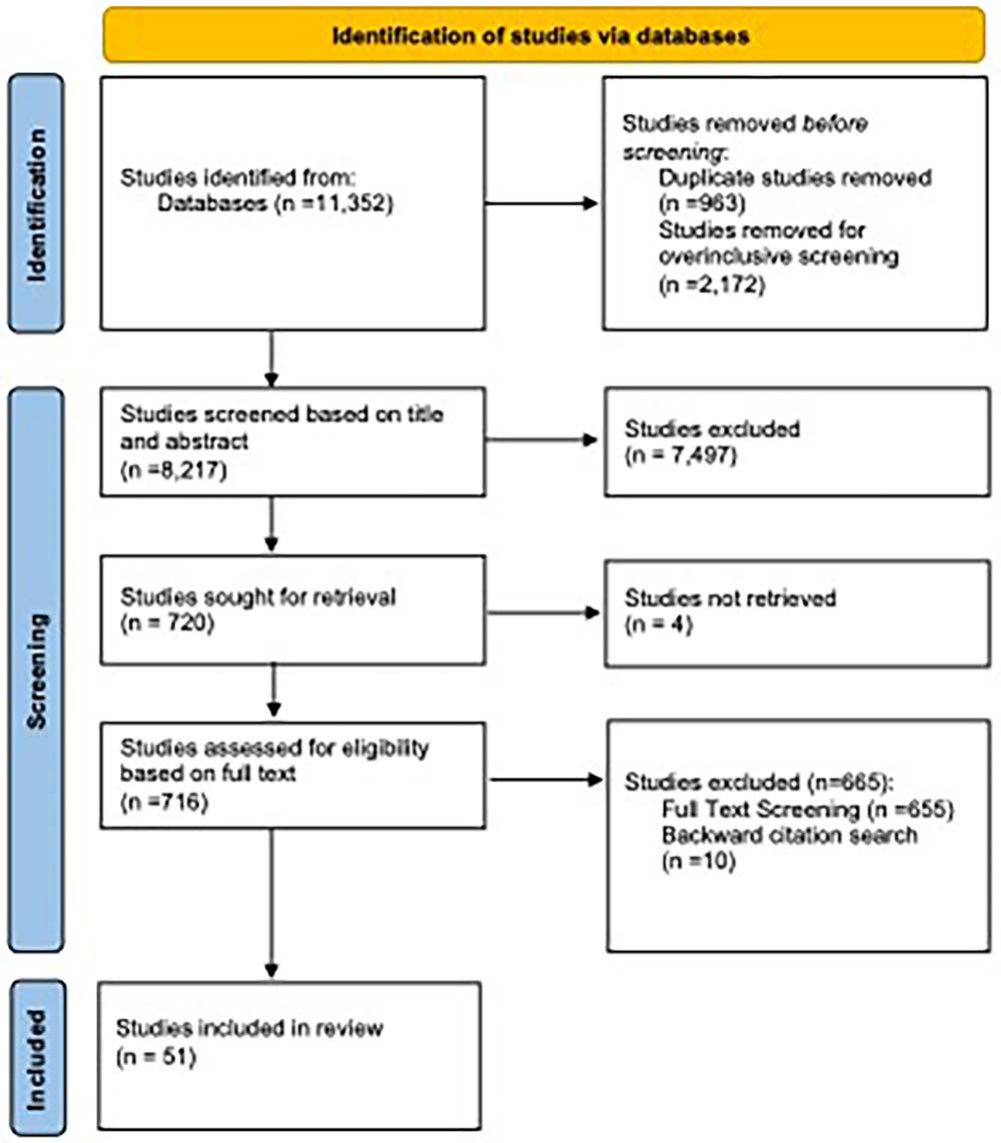
PRISMA flow diagram.

The search output consisted of 11 352 studies, of which 3135 (27.6%) were removed before screening (Fig. 1). Eight thousand two hundred seventeen (72.3%) were screened at the title and abstract level. Of these, 716 (6.3%) full-text records were assessed for eligibility. Fifty-one (*n* = 51; 0.4%) studies met the inclusion criteria and were included (Fig. 1).

There was substantial heterogeneity of in the design of included studies: implementation studies, retrospective cohort observational studies, case studies, qualitative studies, quality improvement reports, and reviews. Of the 51 studies included in the review, 49 described either single studies, or collated data from several similar models (see Supplementary File S3 for details of included studies), and two were reviews (Englander et al. 2022, Fathma et al. 2025).

From the 49 primary studies, four distinct models were identified (Table 1). Studies were mapped according to their scope, focus of care, implementation setting, and specific care components. The graphical representation of the identified models (see Supplementary File S4) emphasizes the significant differences between them in how they link with other services once patients are discharged, and the focus on either high volume (non-specialist) care or target the most complex patients for more person-centred care.

**Table 1:** Core characteristics of the models identified.

**Models: core characteristics**					
**Type**	**Definition and aim**	**Scope**	**Focus of care delivery**	**Implementation setting**	**Number of studies (%)**
**Consultation liaison** (Fleming et al. 1995, Zacharias et al. 1998, Elliott et al., 2003, Rochat et al. 2004, Williams et al. 2005, Ryder et al. 2010, Mcpherson and Benson 2011, Moriarty 2011, Englander et al. 2019, Braithwaite et al. 2021, Dorey et al. 2021, Elphinston et al. 2021, Wilson et al. 2022, Bernal-Sobrino et al. 2023, Mahle et al. 2023, Singh-Tan et al. 2023, Abiri et al. 2024, Quelch et al. 2024, Akhlaghi et al. 2025, George et al. 2025, Lambert et al. 2025)	Small liaison team facilitating MDT support and training; referred complex cases, range of interventions delivered/advised as well as staff training	Targeted	Specialist team	Inpatient/acute	21/49 (42.8%)
**SBIRT** (Welte et al. 1998, Désy and Perhats 2008, Bernstein et al. 2009, Spence et al. 2009, Zimmermann et al. 2018, Van Der Westhuizen et al. 2019, Hays et al. 2020, Avalone et al. 2024, Tryggedsson et al. 2024)	Top down, organization/service wide model delivered per protocol by non-specialist staff. Mainly USA	Broad	Non-specialist	Inpatient/acute	9/49 (18.3%)
**Protocol implementation** (Melson et al. 2014, Wei et al. 2015, Leuenberger et al. 2017, Anderson et al. 2021, Arms et al. 2022, Claus 2022, Kools et al. 2022, Luzum et al. 2024, Calcaterra et al. 2025, Cole et al. 2025)	Locally driven service improvement with aim to standardize management of some parts of patient pathway by non-specialist staff	Narrow	Non-specialist	Inpatient/acute	10/49 (20.4%)
**Supported diversion** (Sorsdahl et al. 2012, Hughes et al. 2013, Fincham-Campbell et al. 2018, Corace et al. 2019, Quelch et al. 2019, Wiercigroch et al. 2020, Anderson et al. 2023, Campbell et al. 2025, Mylonas et al. 2025)	Aim to facilitate rapid, supported discharge to specialist services, often targeted to specific high need groups	Targeted	Specialist team	Outpatient/community	9/49 (18.3%)

MDT, multidisciplinary team; SBIRT, screening, brief intervention, and referral to treatment.

Most studies were conducted in high-income countries: the USA (*n* = 28/49, 57%), UK (*n* = 11/49, 22%), Canada (*n* = 2/49, 4%), Australia (*n* = 2/49, 4%), Denmark (*n* = 1/49, 2%), Spain (*n* = 1, 2%), Switzerland (*n* = 1, 2%), and Netherlands (*n* = 1, 2%). Two studies (Sorsdahl et al. 2012, Van Der Westhuizen et al. 2019) were conducted in South Africa, a middle-income country (*n* = 2, 5%).

Across the models, a wide range of alcohol-associated presentations were addressed, from screening for levels of alcohol consumption in trauma centres, to case management of severe AUD, and alcohol related liver disease. Models of care were variably operationalized to be delivered by specialist staff (e.g. addiction medicine, specialist nurses or social workers, peer navigators, hepatology, or psychiatry teams) or non-specialist teams as part of general medical or surgical care. All studies identified patients within general hospital settings (including emergency departments), and most components were primarily implemented there, but some were delivered in outpatient facilities or community settings. Only six studies reported the involvement of experts by experience as part of their model (Englander et al. 2019, 2022, Wilson et al. 2022, Singh-Tan et al. 2023, Avalone et al. 2024, Akhlaghi et al. 2025). Specialist input for the management of mental health comorbidities was reported in a total of 11 studies, usually within a consultation liaison model (Fleming et al. 1995; Zacharias et al. 1998; Rochat et al. 2004; Moriarty 2011; Hughes et al. 2013; Corace et al. 2019; Englander et al. 2019; Kools et al. 2022; Bernal-Sobrino et al. 2023; Akhlaghi et al. 2025).

### Consultation liaison models

As a patient-oriented model, consultation liaison aims to strengthen multidisciplinary collaboration and links between services in complex clinical systems (Grover and Naskar 2023). Consultation liaison models included in this review all described specific services and were primarily implemented in high-income countries (Supplementary File S3). They provided specialist multidisciplinary support, advice, and assistance on the management and care of patients with AUD, they prioritized patients with a known AUD or those with an alcohol-associated admission. Most provided hospital-wide coverage, but some restricted their operation to clinical areas with a high prevalence of AUD (Roberts et al. 2019), involving settings such as ED (Williams et al. 2005, Elphinston et al. 2021), gastroenterology (Elliott et al., 2003), and surgical wards (Englander et al. 2019), and one specifically reported on an integrated model with hepatology (Mahle et al. 2023).

Only five consultation liaison studies (23%) reported systematic screening for AUD (Fleming et al. 1995; Zacharias et al. 1998; Williams et al. 2005; Mcpherson and Benson 2011; Lambert et al. 2025), relying instead on clinical referral, with the implication that a consultation liaison model without systematic screening will only ever reach a small (and potentially more severe) proportion of patients with AUD in the hospital.

BI/BA was identified as a component of care in nine studies (Fleming et al. 1995; Zacharias et al. 1998; Williams et al. 2005; Ryder et al. 2010; Mcpherson and Benson 2011; Moriarty 2011; Elphinston et al. 2021; Wilson et al. 2022; George et al. 2025), with most teams also supporting the provision of MAAW (Fleming et al. 1995; Zacharias et al. 1998; Elliott et al., 2003; Rochat et al. 2004; Ryder et al. 2010; Mcpherson and Benson 2011; Moriarty 2011; Englander et al. 2019; Dorey et al. 2021; Elphinston et al. 2021; Wilson et al. 2022; Bernal-Sobrino et al. 2023; Singh-Tan et al. 2023; Quelch et al. 2024); in two, this was provided on an outpatient basis (Bernal-Sobrino et al. 2023, Quelch et al. 2024).

Seven facilitated the initiation of RP treatment (Dorey et al. 2021; Wilson et al. 2022; Mahle et al. 2023; Singh-Tan et al. 2023; Abiri et al. 2024; Lambert et al., 2025). Most consultation liaison offered additional psychosocial support (Table 2), including the delivery of motivational interviewing (MI) (Englander et al. 2019, Braithwaite et al. 2021, Wilson et al. 2022, Bernal-Sobrino et al. 2023), CBT (Mcpherson and Benson 2011, Englander et al. 2019), psychoeducation (Zacharias et al. 1998; Englander et al. 2019), and mediation of peer mentoring (Englander et al. 2019, Wilson et al. 2022, Singh-Tan et al. 2023, Akhlaghi et al. 2025).

**Table 2:** Components of care for studies classified as consultation liaison models (*n* = 21).^b^

**Authors (year)**	**Systematic screening**	**BI/BA**	**MAAW**	**RP initiation**	**Additional psychosocial interventions** ^**a**^	**Transition to community at discharge**	**Ongoing training provision to other teams**
Abiri et al. (2024)				X		X	
Akhlaghi et al. (2025)					Peer support, housing support	X	
Bernal-Sobrino et al. (2023)			X		MI	X	
Braithwaite et al. (2021)					MI plus counselling	X	X
Dorey et al. (2021)			X	X	Orientation towards recovery	X	X
Elliott et al. (2003)			X			X	X
Elphinston et al. (2021)		X	X			X	X
Englander et al. (2019)			X		MI, psychoeducation, CBT, step work, family work, peer mentoring	X	X
Fleming et al. (1995)	X	X	X		Family support	X	X
George et al. (2025)		X		X		X	
Lambert et al. (2025)	X		X	X	X	X	
Mahle et al. (2023)				X	X		
Mcpherson and Benson (2011)	X	X	X		CBT, health education, and promotion	X	X
Moriarty (2011)		X	X		Relapse prevention work and education, health promotion	X	X
Quelch et al. (2024)			X		Harm reduction advice, RP techniques (as outpatient)	X	X
Rochat et al. (2004)			X			X	
Ryder et al. (2010)		X	X			X	
Singh-Tan et al. (2023)			X	X	Peer engagement	X	X
Williams et al. (2005)	X	X					X
Wilson et al. (2022)		X	X	X	MI	X	
Zacharias et al. (1998)	X	X	X		Psychoeducation of patient and family	X	

BA, brief advice; BI, brief intervention; CBT, cognitive behavioural therapy; MAAW, medically assisted alcohol withdrawal; MI, motivational interviewing; RP: relapse prevention.

^a^As described by the study authors.

^b^Components Englander et al. (2022) not extracted in the table as this is a review detailing a taxonomy of models.

Nevertheless, only six studies (27%) reported liaison with mental health care for the management of mental health comorbidities (Fleming et al. 1995; Zacharias et al. 1998; Rochat et al. 2004; Moriarty 2011; Englander et al. 2022; Bernal-Sobrino et al. 2023; Akhlaghi et al. 2025). Three embedded family-oriented approaches as part of their model (Fleming et al. 1995; Zacharias et al. 1998; Englander et al. 2019). All studies but one (Williams et al. 2005) an early UK study based in ED, facilitated the transition between acute hospital and community treatment settings.

In contrast with other types of service, a core role of consultation liaison teams involved training general hospital staff (Fleming et al. 1995; Elliott et al., 2003; Williams et al. 2005; Mcpherson and Benson 2011; Moriarty 2011; Englander et al. 2019; Braithwaite et al. 2021; Dorey et al. 2021; Elphinston et al. 2021; Singh-Tan et al. 2023; Quelch et al. 2024).

Seven liaison models based in UK hospitals described a specific variant of an alcohol consultation liaison team, namely an ACT (Elliott et al., 2003; Williams et al. 2005; Ryder et al. 2010; Mcpherson and Benson 2011; Moriarty 2011; Dorey et al. 2021; Quelch et al. 2024). These evolved from early local initiatives to employ an ‘alcohol nurse’ in areas which managed a significant number of patients presenting with alcohol related harm (e.g. ED, general medicine, or Gastro/Hepatology wards), into more structured teams, delivering patient care, and staff training. As with all the consultation liaison models reported, they have significant heterogeneity of scope and are usually established by local clinical champions.

### Screening, brief intervention, and referral to treatment models

Screening, brief intervention, and referral to treatment (SBIRT) is a structured, public health approach aimed at identifying, reducing and preventing alcohol-related harm, with non-specialist staff delivering SBIRT for unscheduled admissions (Madras et al. 2009). Seven SBIRT studies were conducted in the USA (Welte et al. 1998, Bernstein et al. 2007, Désy and Perhats 2008, Spence et al. 2009, Zimmermann et al. 2018, Hays et al. 2020, Avalone et al. 2024), one in Denmark (Tryggedsson et al. 2024), and one study in a low-resourced setting (Van Der Westhuizen et al. 2019). Most SBIRT models were either implemented in ED (Bernstein et al. 2007, Désy and Perhats 2008, Van Der Westhuizen et al. 2019, Avalone et al. 2024) or trauma centres (Spence et al. 2009, Zimmermann et al. 2018, Hays et al. 2020), although one study examined the implementation of SBIRT across multiple settings, including community health clinics (Spence et al. 2009). Where specified, SBIRT was delivered 24 h/day, 7 days a week (Supplementary File S3).

All SBIRT studies reported systematic screening, BI/BA, and referral to community treatment as central components of care (Table 3). Systematic screening was conducted using validated screening questionnaires or by measuring blood alcohol concentration. Two SBIRT models described additional components: MAAW, RP treatment initiation and counselling and MI (Avalone et al. 2024), and problem-solving therapy (Van Der Westhuizen et al. 2019). Transition from hospitals entailed the referral to specialist services, placement in formal treatment programs, and assistance in arranging appointments (warm referrals). The provision of ongoing training to other teams was limited (Welte et al. 1998, Avalone et al. 2024).

**Table 3:** Components of care for studies classified as SBIRT (screening, brief intervention, and referral to treatment) models (*n* = 9).

**Authors (year)**	**Systematic screening**	**BI/BA**	**MAAW**	**RP initiation**	**Additional psychosocial interventions** ^**a**^	**Transition to community at discharge**	**Ongoing training provision to other teams**
Avalone et al. (2024)	X	X	X	X	Counselling, MI	X	X
Bernstein et al. (2007)	X	X				X	
Désy and Perhats (2008)	X	X				X	
Hays et al. (2020)	X	X				X	
Spence et al. (2009)	X	X				X	
Tryggedsson et al. (2024)	X	X				X	
Van Der Westhuizen et al. (2019)	X	X			Problem-solving therapy	X	
Welte et al. (1998)	X	X				X	X
Zimmermann et al. (2018)	X	X				X	

BA, brief advice; BI, brief intervention; MAAW, medically assisted alcohol withdrawal; MI, motivational interviewing; RP, relapse prevention.

^a^As described by the study authors.

SBIRT models were feasible to implement within existing workflows in fast-paced settings and involved minimal resources or staff training (Zimmermann et al. 2018, Van Der Westhuizen et al. 2019, Avalone et al. 2024). As they provide a single intervention at presentation, the reporting (and/or provision) of other evidence-based treatment for the holistic management of AUD was limited. SBIRT could be a precursor to more comprehensive care, operating in coordination with supported diversion models and peer support (Sorsdahl et al. 2012, Avalone et al. 2024).

**Table 4:** Components of care for studies classified as protocol implementation models (*n* = 10).

**Authors (year)**	**Systematic screening**	**BI/BA**	**MAAW**	**RP initiation**	**Additional psychosocial interventions** ^**a**^	**Transition to community at discharge**	**Ongoing training provision to other teams**
Anderson et al. (2021)		X		X	X	X	
Arms et al. (2022)			X	X			
Calcaterra et al. (2025)		X		X		X	
Claus (2022)	X		X				
Cole et al. (2025)	X		X				
Kools et al. (2022)			X			X	X
Leuenberger et al. (2017)	X		X				
Luzum et al. (2024)			X	X		X	
Melson et al. (2014)	X		X				
Wei et al. (2015)		X		X		X	

BA, brief advice; BI, brief intervention; MAAW, medically assisted alcohol withdrawal; RP, relapse prevention.

^a^As described by the study authors.

### Protocol implementation models

Protocol implementation models, aimed at implementing standardized, medication-based treatment protocols to optimize MAAW, prevent escalation to intensive care, and facilitate discharge planning, were reported by 10 individual studies-Table 4 (Melson et al. 2014; Wei et al. 2015; Leuenberger et al. 2017; Anderson et al. 2021; Arms et al. 2022; Claus 2022; Kools et al. 2022; Luzum et al. 2024; Calcaterra et al. 2025; Cole et al. 2025). Therefore, this could be conceptualized more as an operational model than a model of care as it involved the execution of standardized treatment guidelines for non-specialist staff across settings where AUD was common: ED (Anderson et al. 2021, Claus 2022), surgical (Leuenberger et al. 2017), gastroenterology and hepatology (Kools et al. 2022) wards, or trauma centres (Calcaterra et al. 2025) (Supplementary File S3).

Although early identification of AUD or risk of severe alcohol withdrawal is essential to successfully initiate treatment protocols, only four reported systematic screening as a component of care (Melson et al. 2014, Leuenberger et al. 2017, Claus 2022, Cole et al. 2025). All reported implementing either MAAW or RP treatment, and one study provided both (Arms et al., 2022). MAAW regimens used validated withdrawal scores to monitor withdrawal and were either fixed-dose (Claus 2022) or symptom-triggered (Melson et al. 2014, Leuenberger et al. 2017). Initiation of RP medication (naltrexone or acamprosate) was reported in nearly half of the studies (Wei et al. 2015; Anderson et al. 2021; Arms et al. 2022; Luzum et al. 2024; Calcaterra et al. 2025). In four of these, discharge planning was coupled with RP treatment initiation (naltrexone) and assistance in transitioning to community services (Wei et al. 2015, Anderson et al. 2021, Luzum et al. 2024, Calcaterra et al. 2025).

As the aim of protocol models was to standardize the implementation of medication protocols, and psychosocial interventions (where present) were restricted to BI/BA delivery (Wei et al. 2015, Anderson et al. 2021). While most studies trained healthcare providers as part of their implementation plan, ongoing training provision to staff was reported by only one study (Kools et al. 2022).

**Table 5:** Components of care for studies classified as supported diversion models (*n* = 9).

**Authors (year)**	**Systematic screening**	**BI/BA**	**MAAW**	**RP initiation**	**Additional psychosocial interventions** ^**a**^	**Transition to community at discharge**	**Ongoing training provision to other teams**
Anderson et al. (2023)		X		X	X	X	
Corace et al. (2019)			X		Yes, not specified	X	
Campbell et al. (2025)		X		X	Motivational interviewing	X	
Fincham-Campbell et al. (2018)					Assertive engagement, mental health support, peer mentoring	X	
Hughes et al. (2013)			X		Psychological support	X	
Mylonas et al. (2025)		X			Assertive engagement, case management, Motivational interviewing, mental health support, other psychological interventions	X	
Quelch et al. (2019)			X		Yes, not specified	X	
Sorsdahl et al. (2012)	X	X				X	
Wiercigroch et al. (2020)		X		X		X	

BA, brief advice; BI, brief intervention; MAAW, medically assisted alcohol withdrawal; RP, relapse prevention.

^a^As described by the study authors.

### Supported diversion models

Diversion models, identified in nine (*n* = 9) studies, were the most heterogenous group (Table 5). They shared a philosophical imperative to move the locus of care from the emergency department or acute hospital, into a specialist treatment setting. Thus, the focus was on facilitating rapid, supported discharge, and easy access to specialist services, with the aim of reducing length of stay and frequency of subsequent alcohol-associated presentations (Sorsdahl et al. 2012; Hughes et al. 2013; Fincham-Campbell et al. 2018; Corace et al. 2019; Quelch et al. 2019; Wiercigroch et al., 2020; Anderson et al. 2023; Campbell et al. 2025; Mylonas et al. 2025).

Three studies, conducted in Canada (Wiercigroch et al. 2020), the UK (Quelch et al. 2019), and the USA (Corace et al. 2019), employed a diversion model reliant on low-barrier, rapid access outpatient services. These provided psychosocial input, both as a BI/BA (Wiercigroch et al. 2020) or extended support (Corace et al. 2019, Quelch et al. 2019). The model involved staff with clinical expertise to assist with supervision of elective MAAW after onward referral from ED. One study also described the provision of timely access to long-term RP medication (Wiercigroch et al. 2020).

Three UK-based Assertive Alcohol Outreach Team (AAOT) studies were included in this category (Hughes et al. 2013, Fincham-Campbell et al. 2018, Mylonas et al. 2025). The aim of AAOT is to improve treatment engagement for people with AUD who present regularly for unscheduled care at general hospitals. It was developed based on the assertive outreach model for mental health care (Drummond et al. 2017), by providing person-centred care for patients with complex comorbidities by bridging the treatment gap through case management and multidisciplinary care in a coordinated approach. Extended psychosocial support (mental health support, peer mentoring) was offered as well as expedited referral and access to MAAW (Hughes et al. 2013, Fincham-Campbell et al. 2018).

Substance use navigators (SUNs), community health workers with training in both addiction and mental health care, were another form of supported diversion in ED settings (Anderson et al. 2023). Following BI/BA, SUNs provided a combination of extended psychosocial support and advice on RP treatment initiation. They facilitated the transition to a low-threshold addiction medicine clinic which offered same-day, in-person, or telehealth support to ensure continuity of care.

### Included scoping reviews of models of care for AUD

Identified by our search of the literature were two other scoping reviews in the area. The first (Englander et al. 2022) combined a scoping review of broader addiction care (*n* = 87 references) with key informant interviews, to construct a taxonomy for describing hospital-based addiction care in the USA. Their taxonomy has significant overlap with our analysis of the literature on management of AUD in acute hospitals. Our ‘consultation liaison’ model, they further subdivide into three categories (‘interprofessional addiction consult service’, psychiatry consult liaison service’, and ‘individual consultant’); their ‘hospital-based alcohol treatment’ model maps to our ‘protocol implementation’, and their ‘in reach’ model (based on a single study with opioid users has some overlap with our ‘supported diversion ‘model’). Where they differ is primarily due to limiting the context to the USA healthcare system, with a broader focus on addictions particularly opioid dependence, and the differences in funding and reimbursement of activity within the US healthcare system.

The second review (Fathma et al. 2025) sought evidence for the concomitant treatment of AUD and alcohol associated liver disease in nontransplant settings. Of the three included reports; two were already included within our ‘consultation liaison’ model (Moriarty 2011, Mahle et al. 2023) and the third focussed on management of outpatients and therefore out of scope of this review.

### Overlap and uncertainties in components in different models of care

Our ability to clearly define all the components of care in each of the individual studies were limited by a number of factors; some papers reported very little specific information (Mahle et al. 2023, Abiri et al. 2024, Akhlaghi et al. 2025); others reported different aspects of the same operational model in different papers (Quelch et al. 2019, 2024, Anderson et al. 2021, 2023) and similarly others alluded to additional input which was integral to the overall provision of care (Mahle et al. 2023), but gave no details of what this entailed. We limited our interpretation of the results of each paper to data given by (or clearly alluded to) by the authors, rather than attempting to extrapolate beyond the data.

However, it is also important to note that the four models described are not mutually exclusive categories; SBIRT can be combined with additional peer support (Avalone et al. 2024) or diversion model (Sorsdahl et al. 2012); consultation liaison can be combined with implementing wider hospital protocols as a core part of their remit; and implementation of a protocol in ED may be combined with a supported diversion element (Anderson et al. 2021, 2023).

## Discussion

The increased rates of alcohol use disorder and alcohol associated harm, most of which is managed in the general hospital setting makes it imperative to define and develop comprehensive and sustainable structures to facilitate evidence-based treatment of patients presenting to general hospital settings with AUD (Kast et al. 2025; Phillips et al., 2025; Sinclair et al. 2025; Williams et al. 2025). Given the lack of a clear evidence base for standardized approaches to address alcohol-associated harm in this setting, this paper presents the first international systematic review of published reports that describe ways that this has been done. Fifty-one (*n* = 51) papers (including two related scoping reviews) were included and from the data abstracted we were able to categories these into four distinct models of care (consultation liaison, SBIRT, protocol implementation, and supported diversion models) according to their defined scope, target population, skill-mix of staff, focus of care, and setting.

### Implementation context

The CICI framework (Pfadenhauer et al. 2017) recognizes interactions between context, implementation and setting, and how these can be operationalized to understand complex interventions at the micro (patient), meso (organizational), and macro (health system) levels. This is particularly important for the effective management of AUD, given that patients are admitted to general hospitals for a brief period of time (<6 days) (Kast et al. 2025). This will be sufficient to put them at risk of significant alcohol withdrawal requiring medical management; and may act as a ‘teachable moment’ (Williams et al. 2005), but in itself is unlikely to be sufficient to facilitate the longer term behavioural change that will have an impact on health outcomes, especially in patients with more severe and longer duration of AUD (Chambers et al. 2021; Kast et al. 2025).

### Clinically driven implementation

In the single case studies included in this review (*n* = 49), it became clear as part of the data collection and abstraction process that each report describes a process that is a response to its context. The authors, primarily clinicians, often explicitly stated that they had responded to a clear clinical need within their own healthcare setting and responded to it, by implementing some form of pathway. These were highly variable depending on the setting in which they worked (e.g. ED, general medicine, hepatology), the provision already in place (screening, consultation liaison, in reach, etc.) and what was feasible within that setting. Feasibility was variously limited by ‘buy-in’ from other staff, how different services were commissioned and/or reimbursed, and whether the aim of the implemented model was to reduce service use (shorter lengths of stay, reduced readmissions) or improve access to evidence-based treatments.

### Health system mandated implementation

The exception to this clinical response to local needs was the SBIRT model. The SBIRT model started as a national recommendation in the USA to bridge primary prevention and treatment services for AUD. SBIRT was then mandated as part of an accreditation process for USA trauma centres and received significant funding to support implementation. The implementation and scalability of SBIRT (once embedded) entails minimal additional resourcing or disruption to existing workflows across health systems (Bernstein et al. 2007, Sorsdahl et al. 2012). In inpatient settings, it enables the delivery of non-specialist care to a broad range of AUD presentations (Phillips et al. 2019) and can facilitate early intervention and reach non-treatment-seeking individuals (Babor et al. 2007, Sorsdahl et al. 2012). SBIRT models may reduce barriers to AUD management and, therefore, address health inequalities. However, in South Africa, where SBIRT was similarly imposed on local emergency departments by regional government, there was ambivalence on the part of emergency department staff, who felt the resources spent on implementing the model could be better deployed on basic medical equipment, of which there was often an inadequate supply (Sorsdahl et al. 2012).

### Healthy system response to alcohol-related harm

To enable us to categories the descriptions given in the literature into distinct, although not mutually exclusive, ‘models’, we focused on the components of care described in the included studies. However, it was clear there were also dynamic aspects contributing to operational variation, namely their interaction with context, implementation, and setting (Pfadenhauer et al. 2017). This disparity can be attributed to various contextual and implementation factors, including historical anomalies, national, and regional priorities, variable local clinical leadership, ongoing stigma, lack of specialist training among general healthcare providers, the assumption that AUD management falls outside the role of general hospital settings, and the consequent lack of funding for those services (Royal College of Psychiatrists 2020; Cazalis et al. 2023; NHS England 2024).

For example, referral and transition to treatment at discharge (regardless of ‘model’ implemented within the hospital setting) are highly contingent upon the availability of services, and processes that are meant to facilitate transition from a general hospital setting to specialist services. There are substantial differences across health systems in how services are provided and reimbursed, resulting in complex pathways which require significant agency on the part of the patient to navigate (Gilburt etal.2015). Operational barriers (referral requirements, hours of opening, geographical access, and public transport) all have significant implications for demonstrating the effectiveness and any attempts to compare outcomes across different models (Hemrage et al. 2024).

Consultation liaison models are designed to integrate specialist expertise into general healthcare practice and are widely employed for the management of other complex long-term conditions, such as diabetes, psychiatry, and neurology (Dalvi et al. 2008). A key component is the inclusion of specialist clinical leadership, which facilitates sustained implementation of protocols, screening, and training (Williams et al. 2025). However, this is more costly than other models and despite the increased rates and complexity of patients presenting with AUD (Phillips et al. 2025), consultation liaison services for AUD are not widely implemented, or sustained (Sinclair et al. 2025).

While the included models emphasize multidisciplinary collaboration, most lacked a formal interface with mental health care. Less than a third of the consultation liaison models reported involvement of mental health professionals as part of their liaison pathway (Fleming et al. 1995; Zacharias et al. 1998; Rochat et al. 2004; Moriarty 2011; Englander et al. 2022; Bernal-Sobrino et al. 2023; Akhlaghi et al. 2025), and this was less in the other models, with only one protocol implementation model and two supported diversion models documenting any input from mental health professionals (Hughes et al. 2013, Corace et al. 2019, Kools et al. 2022). This limited integration represents a substantial treatment gap in hospital-based AUD management, particularly given the intricate and bidirectional relationship between alcohol use, mental ill-health, cognitive decline, and crisis presentations to acute hospitals (Robins et al. 2021; Ummels et al. 2022; Yu et al. 2024).

### Strengths and limitations of this review

This review has a number of strengths. By conceptualizing of a ‘model of care’, which includes several components, rather than focusing on isolated interventions, it provides an insight into the different levels of coordination and integration of pharmacological and psychosocial elements of AUD care as patients flow through general hospital settings. This can inform a shift towards integrated, coordinated care delivery with implications at the individual, treatment, and health system levels.

An additional strength of this review is that it does include a number of ‘meso-level’ components in the model, including systematic screening, training of non-specialist staff, and transition to community at discharge, emphasizing the role of general medical hospitals in AUD treatment pathways for non-treatment seeking populations and the potential to mediate treatment trajectories towards long-term recovery (Kast et al. 2025, Williams et al. 2025). In addition, by considering context, implementation and setting, the present review provides a pragmatic understanding of how these models are delivered in real-world circumstances, supporting the practical applicability of the findings for both research and practice.

Despite the above strengths, there are limitations. The generalizability and transferability of the findings may be primarily applicable to high-income countries, as only two models of care were reported in low-resourced settings (Sorsdahl et al. 2012, Van Der Westhuizen et al. 2019). Secondly, components of care were variably described, and often with very limited detail. Given the lack of evidence regarding the active components that reduce hospital readmissions (Phillips et al. 2025) or the most effective psychosocial intervention to support short acute hospital admissions where physical, mental health comorbidity, and associated cognitive decline are magnified, there remains uncertainty as to what is delivered to patients within these models, or the training and competence of staff to do so (Phillips, Porter and Sinclair, 2020).

The findings of this review may not fully reflect existing care in contexts and settings where hospital-based AUD care is not yet established or impacted by contextual barriers such as cultural norms and stigma (Al-Ghafri et al. 2023). Finally, consistent with previous research, the inherent heterogeneity in clinical purpose, measures, and outcomes across the models precluded an in-depth analysis of their comparative effectiveness or cost-effectiveness (Trowbridge et al. 2017). Therefore, future research should prioritize these aspects, and how they are shaped by context, implementation, and setting.

## Conclusion

This scoping review analysed the existing published models for AUD identification and management in general hospital settings. Four distinct but not mutually exclusive models were identified (consultation liaison, SBIRT, protocol implementation, and supported diversion), which differed in their scope, focus, components of care, and implementation settings. Models of care were shaped by the resources, context, implementation, and setting in which they operated. Consultation liaison models mediated more comprehensive, integrated care, given their inclusion of pharmacological interventions, psychosocial support, clinical leadership, and care coordination elements like community transition. However, an observation across all models was the relative absence of a structured, robust mental health interface, variations in screening practices, and standardized pathways into community services. This highlights a critical gap in current AUD care provision in general hospital settings.

## Supplementary Material

Supplementary_material_agag037

Supplementary_File_5-Interpretation_of_findings_agag037

## Data Availability

The data underlying this article will be shared on reasonable request to the corresponding author.
