# Supplementary material for: Models of care for the management of alcohol use disorder in general hospital settings and transition to the community: a scoping review
Source: Alcohol Alcohol. 2026 Jul 6;61(4):agag037. doi: 10.1093/alcalc/agag037 (PMC13336398; doi:10.1093/alcalc/agag037)
Supplement: Supplementary_material_agag037 [file supplementary_material_agag037.zip › Supplementary_File_2-Search Strategy.docx]

**Supplementary File 2: Search Strategy**

**S1** (MH "Alcohol-Related Disorders+")

**S2** "alcohol use disorder*" OR alcohol*

**S3** AUD

**S4** S1 OR S2 OR S3

**S5** Hospital* OR “emergency room” OR “emergency department”

**S6** (MH "Hospitalization+")

**S7** S5 OR S6

**S8** S4 AND S7

**S9** (MH "Models, Nursing+") OR (MH "Models, Psychological+")

**S10** (MH "Patient Care Management") OR (MH "Patient Care Planning") OR (MH "Advance Care Planning") OR (MH "Advance Directives") OR (MH "Case Management") OR (MH "Critical Pathways") OR (MH "Alcoholism/TH") OR (MH "Alcoholism/DT")

**S11** (MH "Psychosocial Intervention") OR (MH "Early Intervention, Educational") OR (MH "Early Medical Intervention") OR (MH "Crisis Intervention")

**S12** ((medical OR care OR nursing OR nurse OR patient OR psychologi*) N2 (plan* OR manag* OR model* OR intervention*))

**S13** S9 OR S10 OR S11 OR S12

**S14** S8 AND S13
