# Supplementary material for: Models of care for the management of alcohol use disorder in general hospital settings and transition to the community: a scoping review
Source: Alcohol Alcohol. 2026 Jul 6;61(4):agag037. doi: 10.1093/alcalc/agag037 (PMC13336398; doi:10.1093/alcalc/agag037)
Supplement: Supplementary_material_agag037 [file supplementary_material_agag037.zip › Supplementary_File_3-Characteristics of the included studies (n=49), by model.docx]

# Supplementary File 3: Characteristics of the included studies (n=49), by model

| **Authors (Year)** | **Country** | **Funding and COIs** | **Study design and setting** | **Aim of model** | **Target population** | **Staffing** | **Hours of operation** |  |
| --- | --- | --- | --- | --- | --- | --- | --- | --- |
| **Consultation Liaison (n=21)** | | | | | | | | |
| Abiri et al. (2024) | USA | No funding; no COIs | Single-centered pre-and-post intervention period, general hospital setting (n=1) | Improve medication-assisted treatment for AUD and reduce inpatient readmission rates | Patients presenting alcohol withdrawal at any point during their hospital stay | Inpatient pharmacists | Not specified |  |
| Akhlaghi et al. (2025) | Australia | Funded by St Vincent’s Inclusive Health Program 2022; no COIS | Retrospective analysis, emergency department (n=1) | Purpose-built treatment space for patients requiring support for mental health, alcohol or other drug problems | Mental health, alcohol and other drug patients | ED consultants, nurses, psychiatrists and mental health clinicians, addiction medicine specialists, Aboriginal and Torres Strait Islander liaisons, care coordination staff, consumer peer support workers and carer peer support workers | Not specified |  |
| Bernal-Sobrino et al. (2023) | Spain | No funding; no COIs | Cohort, descriptive observational, retrospective study, tertiary teaching hospital (n=1) | Deliver a personalized intervention for alcohol and other substance-related problems | Patients meeting criteria for AUD/other SUDs | Psychiatrists, psychologist, mental health nurse | Not specified |  |
| Braithwaite et al. (2021) | Canada | Funded by Michael Smith Foundation for Health Research, St Paul’s Hospital Foundation, Canadian Institutes of Health Research, Canada Research, University of British Columbia; no COIs | Retrospective observational analysis, university-affiliated acute setting (n=1) | Implement an inpatient addiction medicine consultation service | Patients presenting to hospital with a known SUD | Addiction medicine physicians, social workers, addiction assessment nurse | 7 days a week, 8am-6pm |  |
| Dorey et al. (2021) | UK | Funded by Alcohol Research UK and Collaboration  for Leadership in Applied Health Research  and Care (NIHR CLAHRC); no COIs | Longitudinal qualitative study, general hospital (n=1) | Deliver nurse-led outpatient detoxification | Inpatients and outpatients identified with likely alcohol dependence | Alcohol specialist nurses | Not specified |  |
| Elliott et al. (2003) | UK | Not stated | Audit, acute care hospital (n=1) | Develop and implement an alcohol care pathway to reduce admissions and improve management | Patients admitted to gastroenterology wards with alcohol-related problems | Ward managers, clinical governance coordinator, gastroenterology consultants, service manager, local GP, social worker, pharmacist, risk management member | Not specified |  |
| Elphinston et al. (2021) | Australia | Funding not stated; LH is the Director of Addiction and  Access Services and RE, MW and  AG are employed by the Addiction  and Mental Health Services in Metro  South Health; data preparation and analysis undertaken by external members. | Interrupted time series analysis, ED (n=1) | Implement a consultation-liaison model as part of ED | Patients presenting to ED using alcohol and other drugs or at risk of related harm | Specialist clinical nurse consultants and clinical nurses | 7 days a week, hours not specified |  |
| Englander et al. (2019) | USA | Funded by Oregon Health & Science University; no COIs | Case study, adult medical and surgical wards across an acute care hospital (n=1) | Implement a multicomponent, interprofessional pathway to support hospital-based addiction care | Patients presenting to hospital with a known SUD | Addiction medicine providers (physicians, nurse practitioner, physician assistant), social workers, peer recovery mentors | Not specified |  |
| Fleming et al. (1995) | USA | Not stated | Longitudinal observational study, various inpatient units across university-affiliated acute setting (n=1) | Develop a multidisciplinary, inpatient-based speciality service | Patients screening positive for SUD upon admission | Physicians, alcohol and drug counsellors, psychiatric nurse specialist, staff assistant | 24 hours, 7 days a week |  |
| George et al. (2025) | USA | Funding not stated; MM owns stock and is employed by Amygdala Neuroscience, consults for GlaxoSmithKline, HepaTX, Prodigy and received grants from Durect Corporation | Retrospective cohort study, academic medical centres (n=2) | Integrate hepatology with addiction medicine for hospitalized patients with AUD | Patients hospitalized with a diagnosis of AUD | Hepatology and inpatient addiction medicine teams | Not specified |  |
| Lambert et al. (2025) | USA | Funding not stated; SW is an author for UpToDate and a textbook editor for Wolters Kluwer and Springer on topics related to addiction | Retrospective cohort study, academic medical centre (n=1) | Increase hospital-based SUD treatment | Patients with an SUD | Addiction medicine physicians, nurse practitioners, social workers, recovery coaches, addiction medicine fellows | Not specified |  |
| Mahle et al. (2023) | USA | Not stated | Single-centre, prospective stud, general hospital setting (n=1) | Dedicated inpatient alcohol liver evaluation team for patients with AUD and ARLD for patients admitted to the hospital for non-liver related complaints | Patients with AUD admitted to hospital with no known liver disease or evidence of current liver disease | Hepatologist and nurse practitioner | Not specified |  |
| McPherson et al. (2011) | UK | Not stated | Observational cohort study, inpatient setting across acute hospitals (n=6) | Nurse-led acute addiction liaison service aimed at supporting links between acute hospitals and community addiction services | Patients presenting to hospital with a known SUD | Nurse team leader, senior addiction nurses trained in general and mental health | Not specified |  |
| Moriarty et al. (2011) | UK | Funding not stated; KM is the Alcohol Services Lead  of the British Society of Gastroenterology and an executive member of the Alcohol Health Alliance UK. | Case study, acute care hospital (n=1) | Provide ward-based, consultant-led multidisciplinary care | Patients presenting to hospital with an alcohol-related admission | Consultant gastroenterologists, specialist liaison psychiatrist, liver nurse practitioner, gastroenterology ward nurses and social worker | 7 days a week, hours not specified |  |
| Quelch et al. (2024) | UK | Funding not stated; AC received funds from Grünenthal (pharmaceutical company) | Case study, acute care hospital (n=1) | Identify, intervene, support discharge and transition to community treatment | Patients presenting to hospital with an alcohol-related admission | Medical toxicology consultant, alcohol nurse consultant, alcohol nurse lead, alcohol specialist nurse, alcohol practitioner, service administrator | 24 hours a day, 7 days a week |  |
| Rochat et al. (2004) | Switzerland | Funded by the Swiss Federal Office of Public Health; COIs not stated | Prospective cohort study, university-affiliated general primary hospital (n=1) | Provide a standardized evaluation and referral process | Patients presenting to hospital with AUD | Internists, psychiatrist, social worker | Not specified |  |
| Ryder et al. (2010) | UK | Not stated | Audit, university-affiliated hospital (n=1) | Provide input for patients with alcohol-related physical disease and support transition to community services | Inpatients identified as having an alcohol-related problem | Registered nurses | Not specified |  |
| Singh-Tan et al. (2023) | USA | Funded by the National Institutes of Health, National Center for Advancing Translational Science and Health Resources and Services Administration; no COIs | Retrospective propensity score-matched historical control design, university-affiliated hospital (n=1) | Provide evidence-based SUD treatment, addressing complex medical decision making, patient engagement and focus on harm reduction | Inpatients with AUD | Addiction medicine physician, addiction medicine fellow, peer advocate, medical trainees | Not specified |  |
| Williams et al. (2005) | UK | Funded by the Alcohol Education and Research Council; COIs not stated | Retrospective cohort study, ED setting (n=1) | Provide prompt follow-up following presentation to ED | Inpatients screening positive for AUD | ED staff, alcohol health worker | Not specified |  |
| Wilson et al. (2022) | USA | Funded by the National Institute on Drug Abuse and National Heart, Lung and Blood Institute | Propensity score-matched case-control study, university-affiliated hospital | Provide an inpatient addiction medicine consultation service | Patients presenting to hospital with a known SUD | Physician, registered addiction nurse, licensed social worker, peer navigator with lived experience | Not specified |  |
| Zacharias et al. (1998) | USA | Not stated | Review, various wards across an acute setting (n=1) | Provide an interdisciplinary approach to developing and implementing a clinical pathway for alcohol withdrawal | Patients presenting to hospital with an alcohol-related problem | Neurosurgeon, psychiatric physician, general medicine physician, specialist in critical care and pulmonary medicine, clinical pharmacist, medicine case manager, ICU staff nurse, substance use intervention nurse, specialist critical care clinical nurse | Not specified |  |
| **SBIRT (n=9)** | | | | | | | | |
| Avalone et al. (2024) | USA | No funding; no COIs | Descriptive study, EDs (n=3) | Integrate SBIRT and peer support in ED | ED patients screening positive for risky substance use or SUD | Social workers and peer counsellors | Site 1: 7 days a week, 8:30am-4:30pm  Site 2: Monday-Friday, 5am-9pm, weekend 5am-1pm  Site 3: Monday-Friday, 6:30am-4:30pm |  |
| Bernstein et al. (2007) | USA | Funded by National Institute on Alcohol Abuse and Alcoholism (NIAAA); no COIs | Quasi-experimental comparison group design, EDs (n=14) | Implement ED provider-initiated SBIRT to reduce alcohol intake | ED patients exceeding low risk drinking guidelines | ED staff (physician, nurse/nurse practitioner, social worker, emergency medical technician) | 24 hours, 7 days a week |  |
| Désy et al. (2008) | USA | Funded by National Highway Traffic Administration (NHTSA); COIs not stated | Prospective study, EDs (n=5) | Implement nurse-led delivery of SBIRT in ED | ED patients at-risk for an alcohol use problem | ED nursing staff | 24 hours, 7 days a week |  |
| Hays et al. (2020) | USA | Funding not stated; no COIs | Feasibility study/program implementation, Level 1 trauma centre (n=1) | Implement SBIRT in a Level 1 trauma centre | Trauma patients with an elevated blood alcohol concentration | Behavioural Medicine clinicians | Not specified |  |
| Spence et al. (2009) | USA | Funded by the Texas Department of State Health Services (DSHS); COIs not stated | Retrospective analysis, multiple settings including trauma centres, internal medicine services and community health clinics (n=6) | Implement SBIRT in a large, urban, publicly funded healthcare system | Patients screening positive for alcohol and drug use upon admission | Physicians, nurses, nurse practitioners, physician assistants, patient care technicians | Not specified |  |
| Tryggedsson et al. (2024) | Denmark | Funded by the Tryg Foundation, Lundbeck Foundation, University of Southern Denmark, Region of Southern Denmark | Randomized controlled trial, one general hospital setting (n=1) and one emergency department (n=1) | Delivery of SBIRT by an employee from the specialized alcohol treatment to increase engagement post-discharge | Patients admitted to hospital with an AUDIT score of 8+ | Employee from the specialized alcohol treatment | Not specified |  |
| van der Westhuizen et al. (2019) | South Africa | Funded by DELTAS Africa Initiative, African Academy of Sciences, Wellcome Trust, Western Cape Office of the Premier; no COIs | Mixed methods sequential explanatory design, emergency centres (n=3) | Implement task-shared SBIRT in low-resourced settings | Patients screening positive for risky substance use | Health counsellors (non-professional healthcare workers) | Not specified |  |
| Welte et al. (1998) | USA | Funded by the Robert Wood Johnson Foundation; COIs not stated | Quasi-experimental comparison group design, multiple settings including medical-surgical, obstetrics and gynaecology, psychiatric and intensive care units (n=4) | Identify and intervene with hospital patients who have a health risk due to alcohol use | Patients screening positive for alcohol or substance use risk | Physician, nurse, social worker, alcohol counsellor | 24/7 |  |
| Zimmermann et al. (2018) | USA | Funding not stated; no COIs | Program implementation, Level 1 trauma centre (n=1) | Implement SBIRT in Level 1 trauma centre | Trauma patients with an elevated blood alcohol concentration | Social workers | Not specified |  |
| **Protocol implementation (n=10)** | | | | | | | | |
| Anderson et al. (2021) | USA | Not stated | Pilot feasibility program, ED (n=1) | Implement a naltrexone program combined with a brief intervention and substance use navigation in an ED setting | Moderate to severe AUD | ED providers | 24/7 |  |
| Arms et al. (2022) | USA | Funded by UC Davis Health Department of Quality and Safety; no COIs | Quality improvement report, university-affiliated hospital (n=1) | Implement educational interventions and improve the utilisation of medication assisted treatment | Patients admitted to internal medicine teams with an alcohol-related condition with moderate to severe AUD | Physicians, healthcare navigators specialised in SUD treatment | Not specified |  |
| Calcaterra et al. (2025) | USA | Not stated | Multi-methods study design, level 1 trauma centre (n=1) | Reduce negative health outcomes among people with AUD through in-hospital administration of intramuscular naltrexone | Hospital patients with AUD | Addiction consultation service clinicians | Not specified |  |
| Claus et al. (2022) | USA | Not stated | Quality improvement report, community hospital (n=1) | Improve patient screening for risk of alcohol withdrawal and initiation of withdrawal protocol by 25% in ED | Patients at risk of moderate to severe alcohol withdrawal | Nurses, physicians, advanced practice practitioners, pharmacists | Not specified |  |
| Cole et al. (2022) | USA | Funding not stated; DF has links with the Founder of Playbl, KW is listed as the inventor of a machine-learning-based risk prediction model | Prospective observational cohort study community hospitals, large complex medical centres and tertiary academic centres (n=7) | Implement an integrated clinical pathway to increase initiation of AUD medication during hospitalization | Adult inpatients diagnosed with AUD and withdrawal | Medical staff | Not specified |  |
| Kools et al. (2022) | Netherlands | No funding; no COIs | Mixed-methods study, gastroenterology and hepatology department at a general hospital (n=1) | Provide an interdisciplinary collaboration approach for hospital patients with AUD | Hospital patients with AUD | Collaboration leader, social psychiatric nurse, gastroenterology nurse, medical social worker, social worker | Not specified |  |
| Leuenberguer et al. (2017) | Switzerland | Funding not stated; no COIs | Evaluation study using a retrospective chart review; ear, nose, throat and jaw surgery department (n=1) | Implement a nurse-led systematic approach to patient screening and alcohol withdrawal management | Patients with an ear, nose, throat or jaw carcinoma with a planned admission | Nurses and medical doctors | Not specified |  |
| Luzum et al. (2024) | USA | Not stated | Quality improvement study, academic hospital (n=1) | Implement evidence-based symptom management for alcohol withdrawal | Inpatients with a discharge diagnosis of alcohol withdrawal | Staff from the hospital medicine, psychiatry, trauma surgery, pharmacy, social work and nursing departments | Not specified |  |
| Melson et al. (2014) | USA | No funding; no COIs | Implementation study, acute care hospital (n=1) | Implement a care management guideline for screening, assessment and symptom management for alcohol withdrawal | Adult inpatients | Nurses, physicians, social worker, pharmacists, data analyst | Not specified |  |
| Wei et al. (2015) | USA | No funding; no COIs | Quality improvement report, university-affiliated hospital | Develop and implement a discharge planning protocol | Patients with alcohol dependence admitted to the internal medicine teaching service | Internal medicine residents | Not specified |  |
| **Supported Diversion (n=9)** | | | | | | | | |
| Anderson et al. (2023) | USA | Funding not stated; no COIs | Implementation study, EDs (n=3) | Implementation of a whole person care-informed intervention delivered by substance use navigators in ED | Patients presenting to ED with an SUD | Substance use navigators (community health workers) | Monday to Friday, 9am-5pm |  |
| Campbell et al. (2025) | USA | Funded by SCELC (Statewide California Electronic Library Consortium) and CA Bridge Program | Quality improvement project, general hospital setting (n=1) | Provide evidence-based, low-threshold treatment for patients with SUD | Hospitalized patients with AUD | Substance use nurse | Not specified |  |
| Corace et al. (2020) | USA | Funding not stated; no COIs | Evaluation study, tertiary mental health care facility (n=1) | Referral to an outpatient rapid access addiction medicine clinic to reduce alcohol-related ED re-utilization and improve quality of care | Patients presenting to ED with an alcohol-related problem | Addiction medicine physicians, nurses, social workers, systems navigator, clinical psychologists and psychiatrists | Monday to Friday, 8am-11am |  |
| Fincham-Campbell et al. (2017) | UK | Funded by the National Institute for Health  Research (NIHR), King’s College London, Health Innovation Network South London, Guy’s and St Thomas’ Charity; no COIs | Cross-sectional national survey, hospitals (n=37)/community | Provide alcohol assertive outreach in the community over long periods and focusing on health care, material resources and coping skills | Patients frequently presenting to hospital with alcohol-related problems | Nurses, community support and drug workers, medical staff, social workers, psychology staff | Not specified |  |
| Hughes et al. (2012) | UK | No funding; No COIs | Retrospective cohort study, acute care hospital (n=1)/community | Provide alcohol assertive outreach in the community through case management and multidisciplinary care | Patients with the highest number of alcohol-related unscheduled care admissions | Consultant in emergency medicine, consultant psychiatrist specialising in addiction disorders, emergency department nurse, social worker, psychologist, alcohol worker, support worker, administrator | Not specified |  |
| Mylonas et al. (2025) | UK | Funded by the NIHR Mental Health Implementation Network and Applied Research Collaboration Greater Manchester | Multistage mixed methods design, assertive outreach services (n=2) | Improve service engagement and healthcare utilization outcomes | Individuals with AUD who face challenges to engaging with community treatment | Nurses, medical doctors, psychologists, community support and drug workers | Not specified |  |
| Quelch et al. (2019) | UK | Funding not stated; no COIs | Retrospective cohort observational study, acute medical unit (n=1) | Provide rapid supported discharge from ED with subsequent elective inpatient detoxification using a symptom-triggered approach and further signposting to community services | Patients admitted to acute medical unit with a discharge diagnosis of alcohol withdrawal | Clinical toxicology consultants and clinical nurse specialists | Not specified |  |
| Sorsdahl et al. (2012) | South Africa | Funded by Department of Social Development of the Western Cape Provincial Government; COIs not stated | Uncontrolled one-group pre- post- outcomes evaluation | Referral to an on-site social worker for SBIRT intervention delivery | Patients with probable substance use | Social worker and auxiliary social worker | Not specified |  |
| Wiercigroch et al. (2020) | Canada | Funded by University Health Network Emergency Medicine Research Fund and University of Toronto; no COIs | Retrospective cohort study, outpatient clinic with links to primary care (n=1) | Improve access to medical treatment for substance use through an outpatient addiction service | Patients with an SUD | Physicians and nurses | Not specified |  |

COI: conflicts of interest; DSHS: Department of State Health Services; ED: emergency department; NHTSA: National Highway Traffic Administration; NIAAA: National Institute on Alcohol Abuse and Alcoholism; SBIRT: screening, brief intervention and referral to treatment; SUD: substance use disorder; UK: United Kingdom; USA: United States of America
