# Supplementary material for: Models of care for the management of alcohol use disorder in general hospital settings and transition to the community: a scoping review
Source: Alcohol Alcohol. 2026 Jul 6;61(4):agag037. doi: 10.1093/alcalc/agag037 (PMC13336398; doi:10.1093/alcalc/agag037)
Supplement: Supplementary_material_agag037 [file supplementary_material_agag037.zip › Supplementary File 4- Graphical representation of the identified models.pdf]

Consultation Liaison

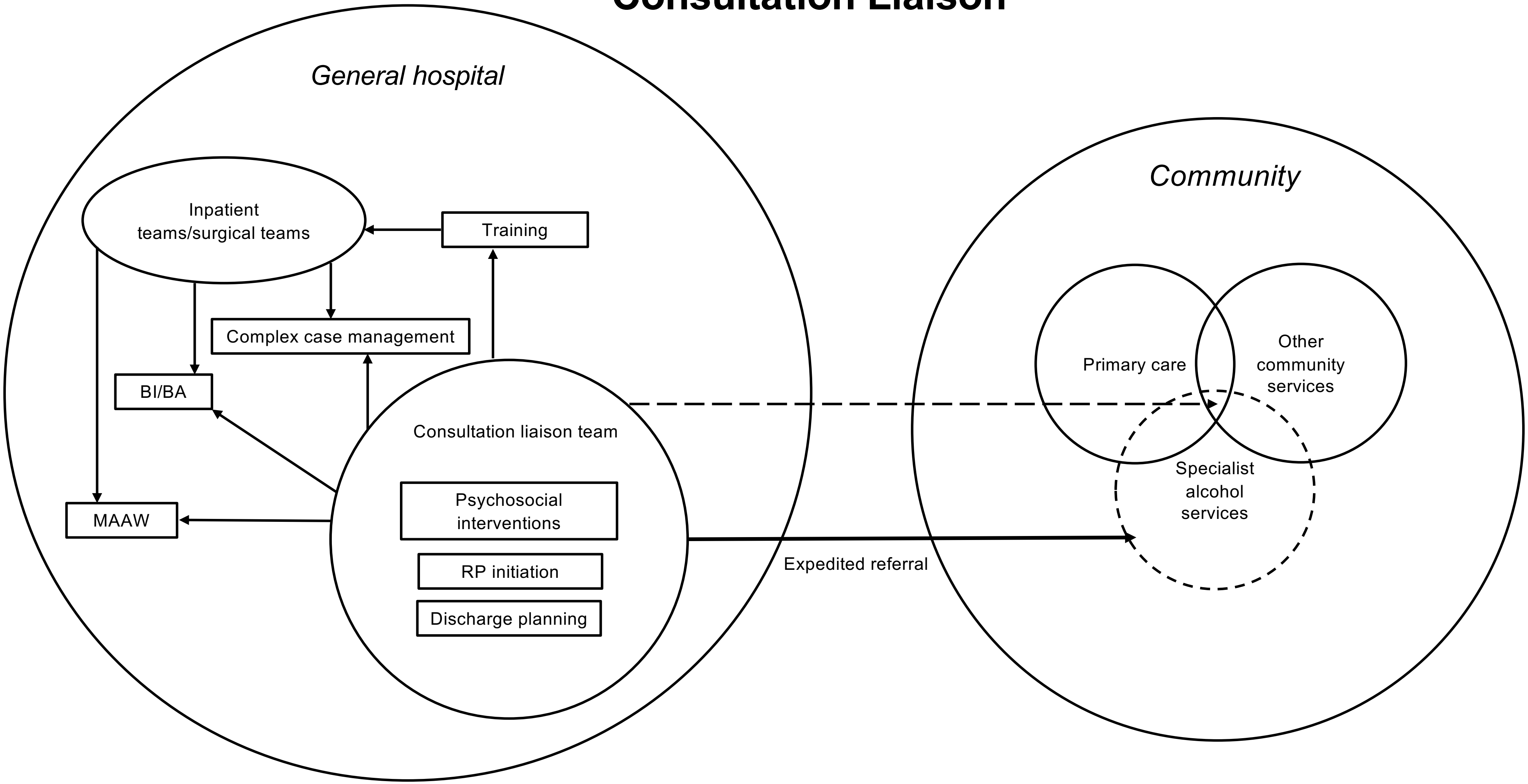

SBIRT

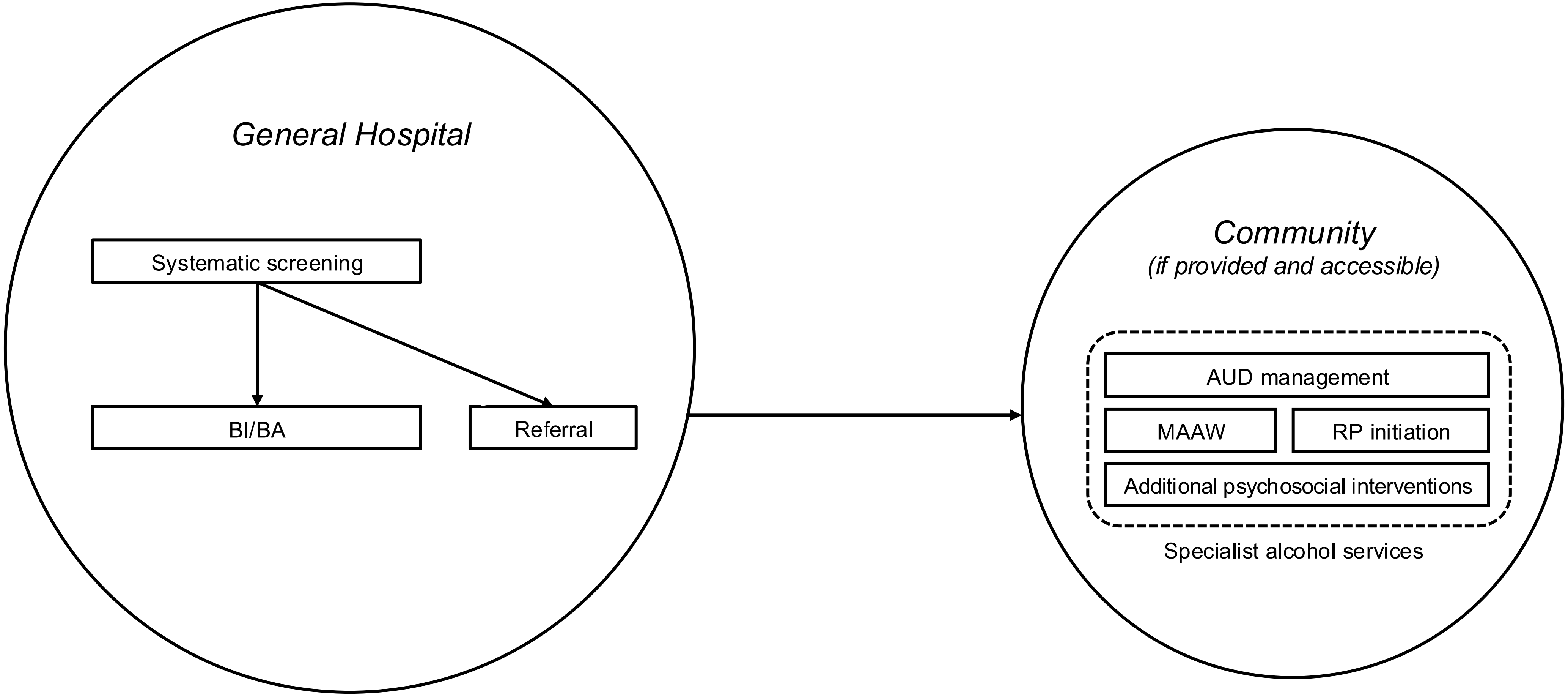
General Hospital
Systematic screening
BI/BA
Referral
Community
(if provided and accessible)
AUD management
MAAW
RP initiation
Additional psychosocial interventions
Specialist alcohol services

Protocol implementation*

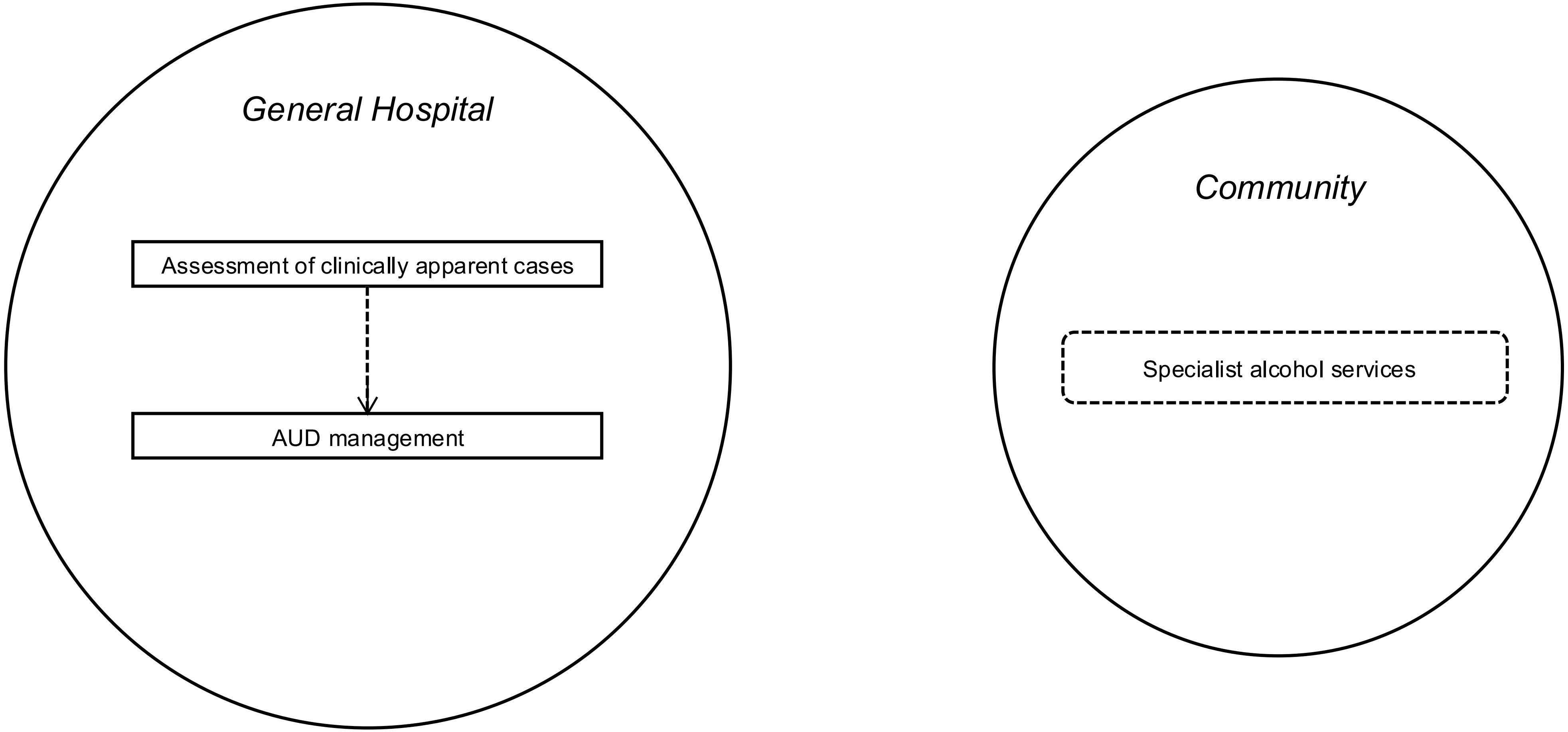

*heterogeneous

Supported Diversion model*

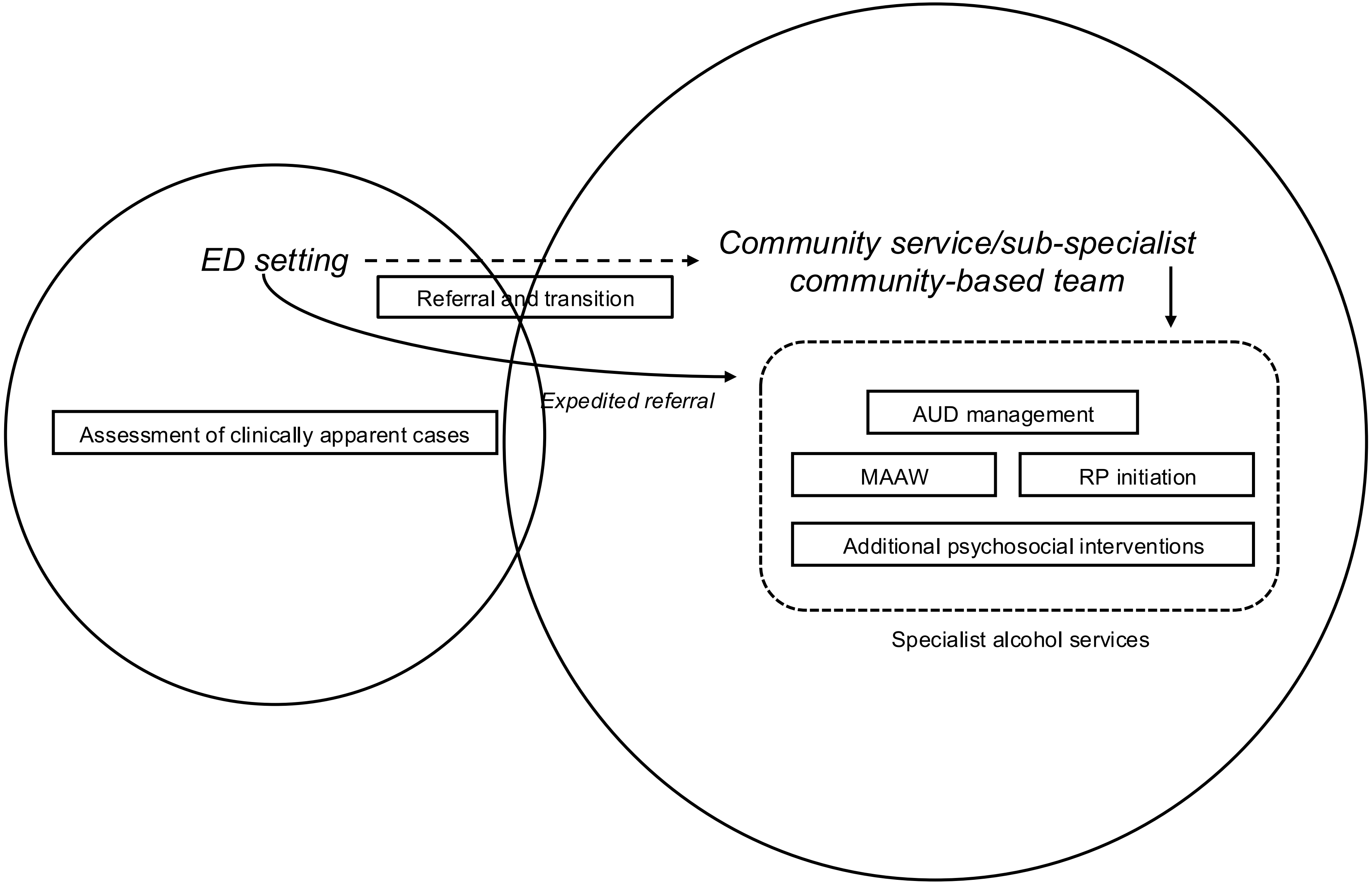

*heterogeneous
