# Supplementary material for: Models of care for the management of alcohol use disorder in general hospital settings and transition to the community: a scoping review
Source: Alcohol Alcohol. 2026 Jul 6;61(4):agag037. doi: 10.1093/alcalc/agag037 (PMC13336398; doi:10.1093/alcalc/agag037)
Supplement: Supplementary_File_5-Interpretation_of_findings_agag037 [file supplementary_file_5-interpretation_of_findings_agag037.docx]

**Interpretation of findings by an expert by experience**

Alcohol use disorder (AUD) is an illness which, for those suffering from it, affects all aspects of their physical and mental well-being daily, and overall quality of life. Mortality rates are high and disproportionately affects those from disadvantaged communities and those with childhood trauma. I lived with this condition for 17 years and know the impact it had on my whole being, and the holistic support required to recover from it. Yet this support is often wanting, with little empathy for those in need of help, and a lack of parity between the approach to AUD and other serious illnesses. This raises the question as to whether it is this lack of empathy, this failure of starting with a philosophy of care, which has led to the inadequate development of treatment pathways. Alcohol patients are stigmatised, often seen as a burden to be sent home as soon as possible. Rather than piecemeal patching up, holistic and comprehensive care integrated with specialist onward support has the potential to aid the person in overcoming their condition thereby benefitting the health system. A holistic approach, including greater screening, earlier intervention, a person-centred treatment plan delivered by well-trained staff, and supported transition to community settings, is needed. Those with lived experience of alcohol addiction are a key piece of this puzzle, in their understanding of the journey to recovery, and are currently underutilised. Bringing together experts by experience and training would be a positive step in further developing the most appropriate model of care.
